# Human Incineration

**Published:** 1918-11-30

**Authors:** 


### HUMAN INCINERATION.

The occurrence of "spontaneous combustion"—such as Dickens' rich imagination seized on and, so to speak, wallowed in, in the death of the alcoholic Krook—we perhaps no longer credit. The medico-legal inquirer when confronted with a solitary burnt corpse probably thinks only of death by accident, of an attempt to conceal the traces of a murder, or of suicide. Strange as it may seem, suicide by burning is not unknown, an instance having been reported quite recently. The easy access to petrol which motoring has caused makes it comparatively easy nowadays to consume the whole of a dead body with the exception, as shown in the disposal of dead soldiers in the present war, of the teeth. A murderer with elbow-room and time at his disposal may therefore attempt by these means to cover up the evidence against him, be it wounds or poison. What looks like such an attempt has been lately reported in the French newspapers; and as they have not scrupled to comment upon the case before its termination, there is obviously no chance, at this distance of space, of justice being interfered with by our doing the same thing. This mysterious and dramatic affair opened with the discovery at Nice of the carbonised remains of a Madame Zon, lying on a partially consumed bed beside which stood an extinguished petroleum lamp. The room was in perfect order except that some dulled, imperfectly transparent panes of glass in a door opening into another room were broken, and on the floor beneath were marks of blood; more blood was found on the bed, under the woman's feet. Medical opinion having been given, and it having been established that the victim was in the habit of curling her hair when in bed, heating the tongs in a lamp placed close by, the magistrates concluded that Madame Zon, when going to bed, had been taken with hæmorrhage followed by a faint, and that the heating apparatus aforesaid then set fire to the bedclothes. A permit of burial was therefore issued.

However, one of the investigators of the occurrence had been struck by the fact that although the corpse had been burnt right down to the feet, the bed was not touched by fire so far down. Also the woman's husband, returned from a visit to Paris, reported that two articles were missing, and two important ones: first, a pearl, a family jewel, worth eighty thousand francs, and secondly, the keys of the room. Further search was made, and in a letter-case in one of the woman's dresses was found the visiting-card of a Serbian officer called Drogolyous, a sub-lieutenant of cavalry. This man, a notorious Don Juan, had been the victim's lover, and on the morning of the discovery had left for Marseilles, making the first part of the journey, as far as Cannes, by tram. The reconstruction of the crime which is offered is that this officer murdered the woman for the pearl, the panes of glass being broken in the struggle; that he then incinerated, first the body and secondly the bed; finally escaping by the side room, locking the door behind him. If this solution prove correct—as to which we say nothing at all, either for or against—the height of the drama was reached, not with the murder at Nice, but at some shop in Marseilles, where, by the way, Drogolyous was arrested when leaving the room of another woman. For the pearl was false, the real one having been sold previously. When taken into custody the accused had in his possession but two hundred francs. It should be added that he energetically denied his guilt.

